# An unusual case of metastasis of a pulmonary undifferentiated pleomorphic sarcoma to the right ventricle: a case report

**DOI:** 10.1186/1752-1947-7-165

**Published:** 2013-06-27

**Authors:** Guodong Xu, Xinbao Shi, Guofeng Shao

**Affiliations:** 1 Department of Thoracic & Cardiovascular Surgery, Lihuili Hospital, Ningbo Medical Center, Affiliated Hospital of Medical School of Ningbo University, Xingning Road, Ningbo 315041, P. R. China

**Keywords:** Cardiac metastasis, Lung undifferentiated pleomorphic sarcoma, Surgical resection

## Abstract

**Introduction:**

Undifferentiated pleomorphic sarcoma is defined as a pleomorphic high-grade sarcoma whose line of differentiation cannot be determined. These tumors constitute less than 5% of all sarcomas in adults. Cardiac neoplasms are rare, and most are metastatic in origin. More than one-third of cardiac metastases originate from lung cancer. Symptoms of cardiac neoplasms usually appear late in the course of the disease and are often ignored because of the more severe effects of the primary malignancy or its therapy. We present the case of a patient with undifferentiated pleomorphic sarcoma of the lung presenting with symptomatic right-heart failure secondary to cardiac metastasis. The purpose of this report is to present this unusual case.

**Case presentation:**

Our patient was a 59-year-old Chinese woman with symptomatic metastasis of an undifferentiated pleomorphic sarcoma of the lung to the right ventricle. She had a history of a stage IV, pulmonary, undifferentiated pleomorphic sarcoma that had been successfully treated with chemotherapy and radiotherapy 4 years ago. A complete response was obtained, and she was in remission until the cardiac metastasis. She underwent surgical excision of the cardiac mass because it caused dyspnea and posed a high risk of sudden death, pulmonary embolism or tricuspid obstruction. Histopathological and immunohistochemical examinations of the surgical specimen established the diagnosis of undifferentiated pleomorphic sarcoma and confirmed that the cardiac tumor was a metastasis from the lung.

**Conclusions:**

In patients who have known metastatic neoplasms and present with cardiac manifestations, whether detected during history taking or physical examination, the clinician should be alert to the possibility of cardiac metastases. In patients with cardiac metastases, the therapeutic alternatives are limited to palliative treatment of symptoms and chemotherapy. In some patients, surgery can be used to relieve symptoms. We have reported the first case of symptomatic cardiac metastases from an undifferentiated pleomorphic sarcoma of the lung. Our patient underwent surgical resection, and her symptoms improved significantly. This case is unique because it is the only reported case of undifferentiated pleomorphic sarcoma of the lung which metastasized to the heart, and in which symptomatic improvement was effectively obtained with surgical resection.

## Introduction

Undifferentiated pleomorphic sarcoma is defined as a pleomorphic high-grade sarcoma whose line of differentiation cannot be determined. These tumors constitute less than 5% of all sarcomas in adults [1]. From a clinical viewpoint, undifferentiated pleomorphic sarcomas are deeply located tumors that show progressive and rapid growth. The mean 5-year survival rate ranges from 50% to 60% [2].

Cardiac neoplasms are rare, and most are metastatic in origin. Cardiac metastases are found at autopsy in 6% to 20% of patients with malignant neoplasms [3]. More than one-third (36%) of cardiac metastases originate from lung cancer. Non-solid primary malignancies such as leukemia, lymphoma and Kaposi sarcoma account for 20% of cardiac metastases, breast carcinoma for 7% and esophageal carcinoma for 6% [4].

Symptoms of cardiac neoplasms usually appear late in the course of the disease and are often ignored because of the more severe effects of the primary malignant disorder or its therapy. Consequently, cardiac neoplasms, especially metastatic ones, are often not discovered until autopsy.

We present the case of a patient with undifferentiated pleomorphic sarcoma of the lung presenting with symptomatic right-heart failure secondary to cardiac metastasis. The purpose of this report is to present this unusual case.

## Case presentation

**Figure 1:**
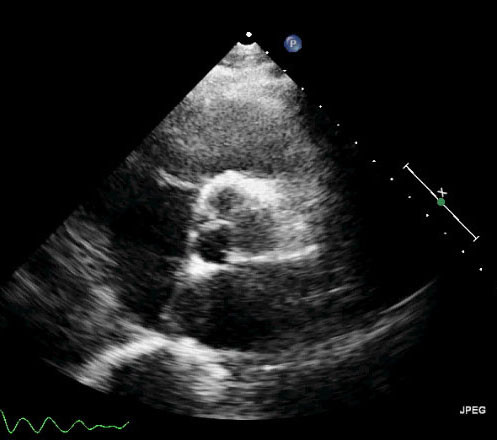
**Transthoracic echocardiographic scan (parasternal short-axis view of aortic root) 4 years ago.** No tumor was revealed in the right ventricle.

A 59-year-old Chinese woman was referred to our hospital 11 months ago because she had had dyspnea for more than 1 month. She had a history of stage IV lung cancer that was located in the left upper lobe. Transthoracic echocardiography revealed no right ventricular tumor at the time the original left lung cancer was diagnosed (Figure 1). The patient was diagnosed by fiberoptic bronchoscopic biopsy. Immunohistochemical analysis demonstrated that the tumor cells were strongly positive for vimentin and CD68, and moderately positive for Ki-67(+). Focal staining for actin and desmin was also observed, but S-100, CD34(-), CK(pan), CAM5.2 and epithelial membrane antigen (EMA) were all negative. Histopathological and immunohistochemical examinations confirmed a diagnosis of undifferentiated pleomorphic sarcoma 4 years ago. She had successfully undergone multiple sessions of radiotherapy and chemotherapy, and no recurrence was observed during follow-up.

On a physical examination, her blood pressure was 110/70mmHg, and her pulse rate was 110 beats per minute and regular. A cardiac examination revealed a right ventricular impulse; a systolic thrill was palpable at the left sternal border, and a grade III/IV systolic ejection murmur was audible in the same region. On auscultation, her chest was clear.

**Figure 2:**
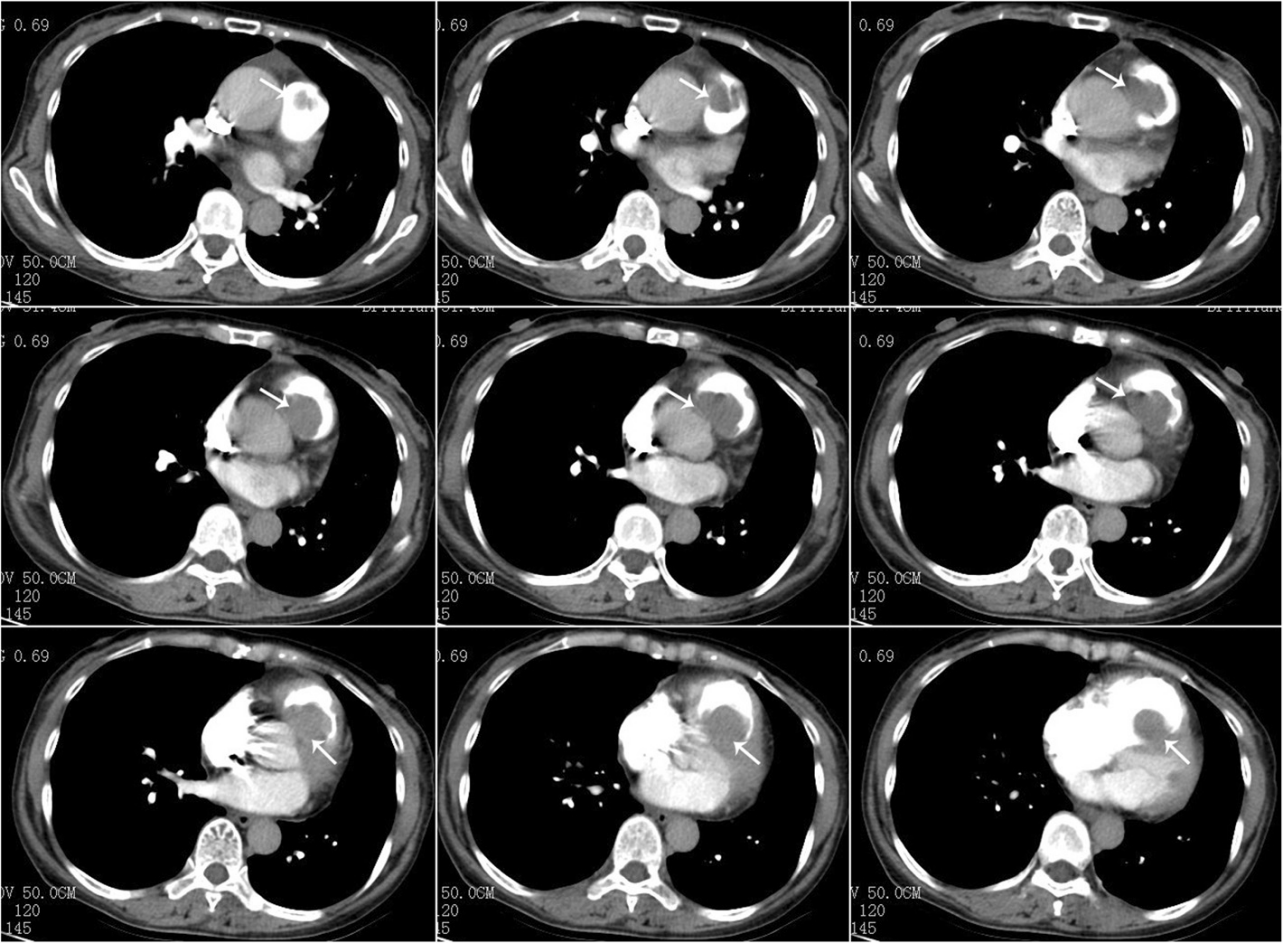
**Preoperative, contrast-enhanced, computed tomographic scan of the chest. **A large mass measuring 5cm × 6cm, filling defects in the main pulmonary artery and right ventricle and hydropericardium can be seen (arrows).

**Figure 3:**
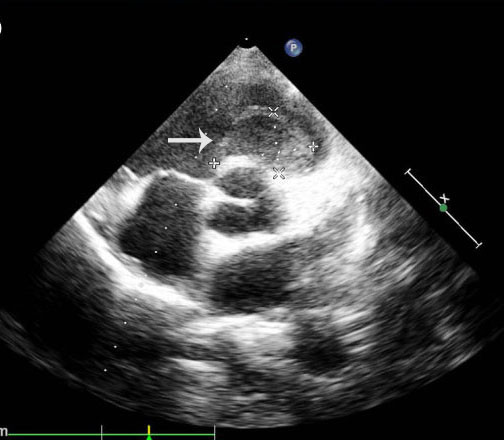
**Preoperative, transthoracic echocardiographic scan (parasternal short-axis view of aortic root). **A tumor measuring approximately 5cm × 5cm and extending from the right ventricle to the pulmonary valve can be seen (arrow).

Contrast-enhanced computed tomography (CT) showed a large mass measuring 5cm × 6cm, filling defects in the main pulmonary artery and right ventricle and hydropericardium (Figure 2). No abnormal findings were seen in the lung. Transthoracic echocardiography revealed enlargement of the right ventricle, hydropericardium and a tumor measuring approximately 5cm × 5cm and extending from the right ventricle to the pulmonary valve (Figure 3). The liver, spleen, pancreas, and kidneys appeared normal. The patient had no evidence of tumor recurrence in the left lung. Her electrocardiogram showed sinus rhythm with no significant ST-T abnormalities. Serum levels of the tumor markers alpha-fetoprotein, carcinoembryonic antigen, carcinoma antigen (CA)-125 and CA 19–9 were not elevated.

**Figure 4:**
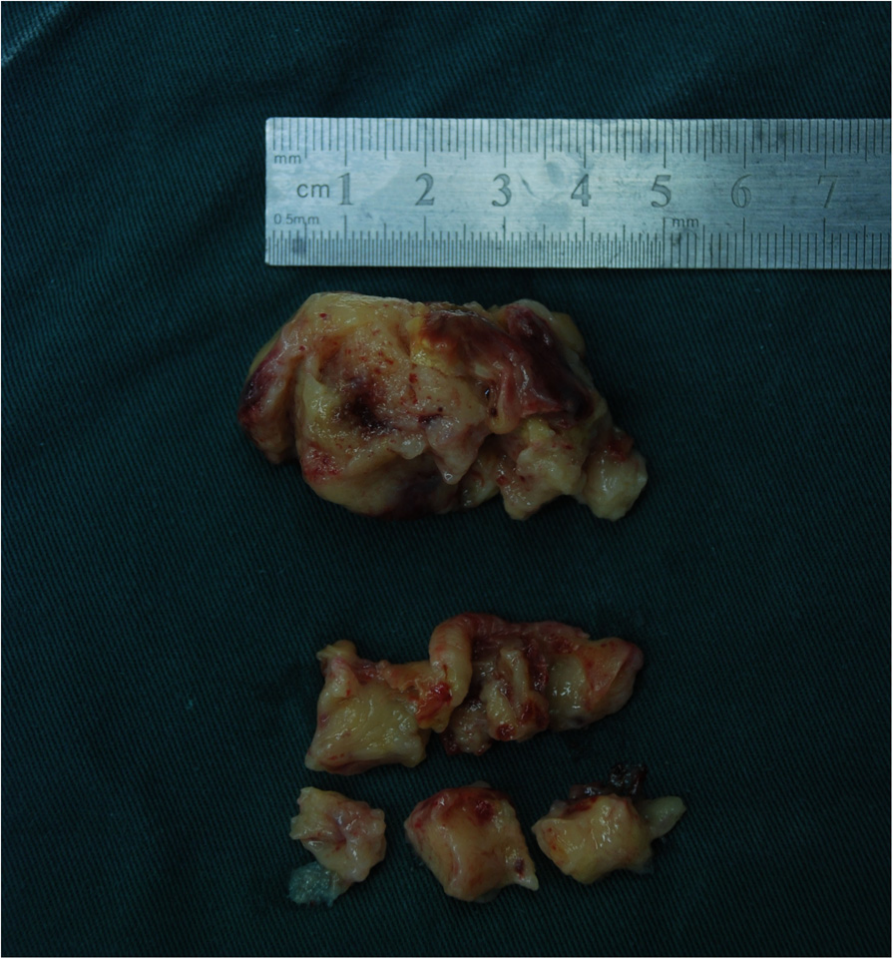
**Intraoperative specimen. **The partially resected tumor obstructed the right ventricular and main pulmonary artery.

**Figure 5:**
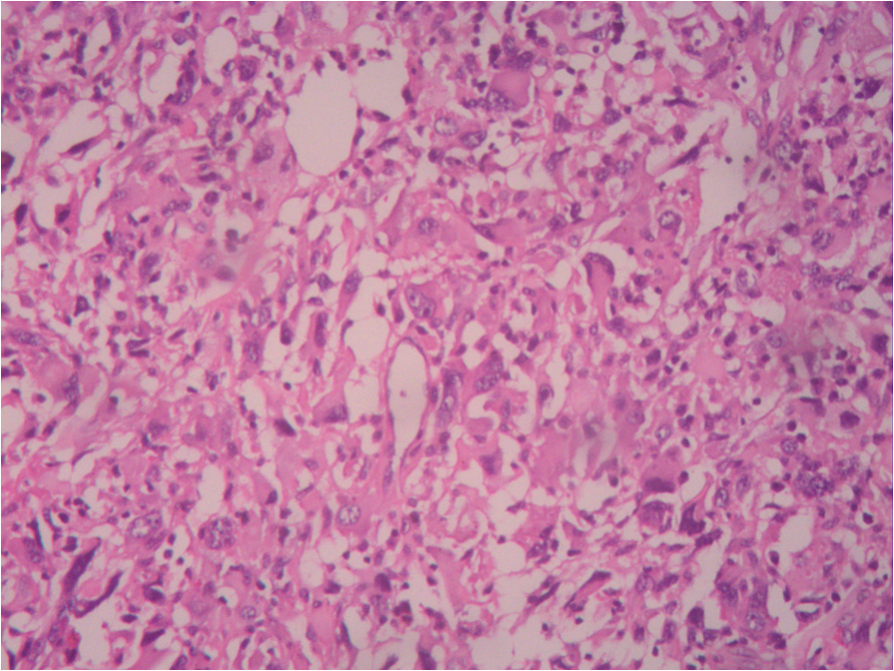
**Photomicrograph of resected specimen. **Histopathological examination of the specimen shows that the tumor is an undifferentiated pleomorphic sarcoma (hematoxylin and eosin stain; original magnification, ×100).

After considering the extent of the patient’s disease, we deemed that an operation was the best alternative. The tumor in the right ventricle and main pulmonary artery was surgically resected; it measured 6cm × 5cm (Figure 4). The postoperative course was uneventful, and she was discharged on day 13 after the surgery. Her dyspnea was significantly relieved postoperatively. Histopathological examination of the resected specimen indicated a diagnosis of undifferentiated sarcoma (Figure 5). Immunohistochemical analysis demonstrated that the tumor cells were strongly positive for vimentin and CD68, and moderately positive for Ki-67(+). Focal staining for actin and desmin was also observed, but S-100, CD34(-), CK(pan), CAM5.2 and EMA were all negative.

When seen at the last follow-up 2 months ago, our patient was in complete remission, with no evidence of tumor recurrence on CT and echocardiography.

## Discussion

We have described the first case of cardiac metastasis from an undifferentiated pleomorphic sarcoma of the lung. Immunohistochemistry may be useful to distinguish pulmonary undifferentiated pleomorphic sarcomas from non-mesenchymal malignant tumors and to delineate the level of differentiation of the sarcomas [5]. The limited data available on undifferentiated pleomorphic sarcomas indicate that this neoplasm has an aggressive clinical course, and a high incidence of recurrence and metastasis. Surgical resection is the primary therapeutic modality of this sarcoma. If the tumor is at an advanced stage, combination chemotherapy or radiotherapy may be used as a palliative approach. The overall 5-year survival of patients with undifferentiated pleomorphic sarcoma is approximately 50% [6]. Our patient was diagnosed with a stage IV, undifferentiated pleomorphic sarcoma of the lung and was treated with multiple cycles of chemotherapy and radiotherapy 4 years ago. This treatment proved efficacious in our patient, and local tumor recurrence did not occur.

Cardiac involvement is not uncommon in lung cancer, and is detected in approximately 25% of autopsy cases [7,8]. In a study by Tamura *et al.*, cardiac involvement was found at autopsy in 23 of 78 patients with lung cancer [9]. Despite the significant mortality and morbidity with which it is associated, cardiac metastasis is usually diagnosed during autopsy. Most cardiac metastases are asymptomatic [10]. Only 10% of patients with cardiac metastasis have symptoms, which are typically related to cardiac function. The clinical presentation varies depending upon the location and extent of myocardial involvement. The small number of reported cases of symptomatic cardiac metastasis attests to the rarity of metastatic lesions that are large enough to cause right ventricular outflow tract obstruction. Our patient presented with dyspnea because of right ventricular outflow tract obstruction. The main differences between primary pulmonary artery sarcoma and metastatic lung disease in the same area is that metastatic lung disease has a history of lung cancer and histopathological and immunohistochemical examinations should be done accordingly with the primary lung cancer. Histopathological and immunohistochemical examinations of the surgical specimen in our patient clearly showed that the cardiac tumor was a metastasis from the lung cancer. Although such cardiac metastases are not uncommon at autopsy, this is the first reported case of a cardiac metastasis from an undifferentiated pleomorphic sarcoma of the lung that was diagnosed in a living patient and was successfully treated with surgery.

Since cardiac metastases are often incurable, surgical resection is not an optimal option for patients with metastatic involvement of the heart. Systemic chemotherapy is usually the most beneficial treatment. Nevertheless, metastatic cardiac involvement poses other problems. Resection of malignant sarcomas is usually only palliative. In cases where the metastasis results in cardiac tamponade or valve obstruction, palliative surgery is often used to relieve symptoms if the primary tumor has been resected *in toto*, and the patient appears to have a good prognosis. Palliative radiotherapy to the heart is rarely helpful or indicated to relieve symptoms.

In patients with unresectable malignant tumor, palliative surgery may be used, but without great expectation of successful results. Bakaeen *et al.*[11] reported that the median survival time of patients with malignant cardiac tumors was 9.6 months. Burke *et al.*[12] reported that the average and median survival in 75 patients with primary malignant cardiac sarcoma was 11 months and 6 months, respectively.

Survival depends on the extent of resection of the sarcomas because of their high potential for recurrence and metastasis. Mean survival after surgical excision has been documented to be 9 to 10 months; a significant predictor of long-term mortality is the presence of New York Heart Association Class III and IV symptoms [11]. The role of adjuvant chemotherapy and radiotherapy is unclear. Given the limited data and the lack of randomized trials, complete surgical resection is currently the only factor that influences survival [13]. In our patient, wide local excision of the recurrent mass was performed, without adjuvant chemotherapy and radiation.

Surgical excision was selected not only because she had had a good outcome after undergoing chemotherapy and radiotherapy 4 years ago but also to prevent intracardiac blood flow obstruction and congestive heart failure. Six months after the surgery, she had recovered well, with no evidence of tumor recurrence on CT and echocardiography.

## Conclusions

Cardiac metastasis from lung cancer is rare and is mainly found at autopsy as an incidental, non-symptomatic finding. In patients who have known metastatic neoplasms and who present with cardiac manifestations, whether detected on history taking or physical examination, the clinician should be alert to the possibility of cardiac metastases. We have reported the first case of symptomatic cardiac metastases from an undifferentiated pleomorphic sarcoma of the lung.

In patients with cardiac metastases, the therapeutic alternatives are limited to palliative treatment of symptoms and chemotherapy. In some patients, surgery can be used to relieve symptoms. Our patient underwent surgical resection, and her symptoms improved significantly. However, treatment of all the metastatic cardiac tumors provides only palliation. Mean survival of affected patients is 3 months to 1 year for metastatic cardiac tumors [14]. The patient will be examined every 3 months by echocardiography in the next 2 years.

This case is unique because it is the only reported case of undifferentiated pleomorphic sarcoma of the lung which metastasized to the heart, and in which symptomatic improvement was obtained after surgical resection.

## Consent

Written informed consent was obtained from the patient for the publication of this case report and the accompanying images. A copy of the written consent is available for review by the Editor-in-Chief of this journal.

## Competing interests

The authors declare that they have no competing interests in the preparation of this article.

## Authors’ contributions

GX drafted the manuscript. GX and XS treated the patient. GS and XS participated in the design of the study. All authors read and approved the final manuscript.
